# Attitudes Toward the Global Allocation of Chinese COVID-19 Vaccines: Cross-sectional Online Survey of Adults Living in China

**DOI:** 10.2196/33484

**Published:** 2022-06-07

**Authors:** Hanzhi Yu, Runming Du, Minmin Wang, Fengyun Yu, Juntao Yang, Lirui Jiao, Zhuoran Wang, Haitao Liu, Peixin Wu, Till Bärnighausen, Lan Xue, Chen Wang, Shannon McMahon, Pascal Geldsetzer, Simiao Chen

**Affiliations:** 1 School of Public Affairs Zhejiang University Hangzhou China; 2 Department of Global Health Peking University School of Public Health Beijing China; 3 Key Laboratory of Carcinogenesis and Translational Research, Laboratory of Genetics, Ministry of Education Peking University Cancer Hospital & Institute Beijing China; 4 State Key Laboratory of Medical Molecular Biology Institute of Basic Medical Sciences Chinese Academy of Medical Sciences and Peking Union Medical College Beijing China; 5 Columbia University New York, NY United States; 6 Chinese Academy of Medical Sciences and Peking Union Medical College Beijing China; 7 Peking Union Medical College Hospital Beijing China; 8 Heidelberg Institute of Global Health Faculty of Medicine and University Hospital Heidelberg University Heidelberg Germany; 9 Department of Global Health and Population Harvard T.H. Chan School of Public Health Boston, MA United States; 10 Africa Health Research Institute Somkhele South Africa; 11 School of Public Policy and Management Tsinghua University Beijing China; 12 National Clinical Research Center for Respiratory Diseases Beijing China; 13 Department of Pulmonary and Critical Care Medicine Center of Respiratory Medicine China-Japan Friendship Hospital Beijing China; 14 Division of Primary Care and Population Health Department of Medicine Stanford University Stanford, CA United States; 15 Chan Zuckerberg Biohub San Francisco, CA United States

**Keywords:** COVID-19 vaccines, China, global allocation, public attitudes, cross-sectional, survey, vaccines, COVID-19, pandemic, public health, health policy, epidemiology

## Abstract

**Background:**

COVID-19 vaccines are in short supply worldwide. China was among the first countries to pledge supplies of the COVID-19 vaccine as a global public product, and to date, the country has provided more than 600 million vaccines to more than 200 countries and regions with low COVID-19 vaccination rates. Understanding the public’s attitude in China toward the global distribution of COVID-19 vaccines could inform global and national decisions, policies, and debates.

**Objective:**

The aim of this study was to determine the attitudes of adults living in China regarding the global allocation of COVID-19 vaccines developed in China and how these attitudes vary across provinces and by sociodemographic characteristics.

**Methods:**

We conducted a cross-sectional online survey among adults registered with the survey company KuRunData. The survey asked participants 31 questions about their attitudes regarding the global allocation of COVID-19 vaccines developed in China. We disaggregated responses by province and sociodemographic characteristics. All analyses used survey sampling weights.

**Results:**

A total of 10,000 participants completed the questionnaire. Participants generally favored providing COVID-19 vaccines to foreign countries before fulfilling domestic needs (75.6%, 95% CI 74.6%-76.5%). Women (3778/4921, 76.8%; odds ratio 1.18, 95% CI 1.07-1.32; *P*=.002) and those living in rural areas (3123/4065, 76.8%; odds ratio 1.13, 95% CI 1.01-1.27; *P*=.03) were especially likely to hold this opinion. Most respondents preferred providing financial support through international platforms rather than directly offering support to individual countries (72.1%, 95% CI 71%-73.1%), while for vaccine products they preferred direct provision to relevant countries instead of via a delivery platform such as COVAX (77.3%, 95% CI 76.3%-78.2%).

**Conclusions:**

Among our survey sample, we found that adults are generally supportive of the international distribution of COVID-19 vaccines, which may encourage policy makers to support and implement the distribution of COVID-19 vaccines developed in China worldwide. Conducting similar surveys in other countries could help align policy makers’ actions on COVID-19 vaccine distribution with the preferences of their constituencies.

## Introduction

Vaccination is a promising approach to achieving global control of COVID-19. It is estimated that over 14 million future deaths could be prevented if COVID-19 vaccines could be delivered sufficiently [1-3]. As of December 31, 2021, over 70% of people in high-income countries are fully vaccinated against COVID-19; while in low-income countries, that number is only 4% [4]. Inequitable allocation of vaccines may lead to avoidable death, social dissatisfaction, and adverse mental health consequences [5,6].

Expanding vaccine coverage to low-income countries is difficult due to insufficient COVID-19 vaccine production and supply; at present, only 22 countries have the capacity to produce COVID-19 vaccines [7]. For most countries, the available vaccines that they can acquire depend on international assistance provided by procurement or purchasing from foreign vaccine companies, bilateral aid between countries, or multilateral aid via institutions (eg, COVAX, a global risk-sharing mechanism for pooled procurement and equitable distribution of COVID-19 vaccines coled by the World Health Organization [WHO], The Global Alliance for Vaccines and Immunizations [GAVI], and the Coalition for Epidemic Preparedness Innovations, with GAVI responsible for vaccine delivery).

Understanding population-level attitudes toward the global allocation of vaccines is important for several reasons. First, international organizations such as the WHO, which coordinates and allocates COVID-19 vaccines at the global level, need to ensure fair allocation and delivery of vaccines with minimal conflicts and dissatisfactions [8-10]. Knowing the public’s attitudes toward the global delivery of vaccines can inform policy making and can support global policy makers to effectively mobilize international communities through their widespread support. Second, public attitudes can have a substantial impact on a country’s national foreign aid policies [11]. National governments may avoid formulating foreign policies that contradict public opinions for political reasons. Third, identifying population groups with high and low support for global COVID-19 vaccine allocation is crucial for developing targeted education and communication campaigns to enhance the recognition and support for a more equitable distribution of vaccines worldwide.

We are not aware of studies exploring public attitudes toward the global allocation of locally produced COVID-19 vaccines (ie, the allocation of COVID-19 vaccines from a domestic population to a global community). Existing research on public attitudes has focused exclusively on domestic allocation of COVID-19 vaccines (ie, priority setting among population groups locally). That literature highlights the public’s broad support for vaccinating medical staff first [12-16]. Within China [12], the United States [13], and Italy [14], further research suggests public support for vaccination among older adults, although a study drawing upon Belgian perspectives found support instead for those who are chronically ill, hold essential professions, or who are most likely to spread the virus [15].

Chinese COVID-19 vaccines manufactured by Sinopharm and Sinovac were listed for emergency use by the WHO in 2021 (on May 7 [17] and June 1 [18], respectively). The WHO Strategic Advisory Group of Experts has thoroughly assessed the data on quality, safety, and efficacy of the vaccine, and has recommended its use for people 18 years and older [19,20]. The immunogenicity and safety of the Chinese recombinant COVID-19 vaccine (adenovirus type 5 vector) codeveloped by CanSinoBIO and the Beijing Institute of Biotechnology have been confirmed through randomized controlled trials [21,22]. The Chinese government was among the first to pledge COVID-19 vaccines as a global public good [23]. As of October 17, 2021, China has provided more than 1.5 billion doses to more than 100 countries and international organizations as donations or as a purchased export [24]. At the same time, China is under intense pressure to meet a sizable domestic demand. To achieve an 80% vaccination rate by the end of 2021, an average of 230 million doses is required each month [25-27].

This study explores the Chinese public’s attitudes toward global COVID-19 vaccine allocation. In doing so, we provide evidence to policy makers in China for formulating future strategies of global COVID-19 vaccine distribution. Furthermore, work such as this can guide similar public surveys in other COVID-19 vaccine-producing countries.

## Methods

### Sampling Process

The survey was implemented by KuRunData, an online private survey platform that maintains a database of potential survey participants and delivers surveys. KuRunData recruits members through its own platform [28] and partnerships with other websites, and by encouraging registered members to recruit new members through the popular mobile app WeChat Mini. KuRunData verifies that members have access to mobile phones and the internet, and are capable of navigating online surveys. For this study, we used KuRunData to sample approximately the same number of participants in each of China’s provincial-level administrative units, with the total sample size goal being 10,000 adults. Potential participants were unable to access the questionnaire once this sample size goal was reached. Within each province, KuRunData aimed to sample a proportion of participants that was reflective of the demographic composition of the province’s population (as per the 2020 China Statistical Yearbook [29]) by sex and urban-rural residence. Adults in the survey pool were invited to participate in the survey by KuRunData’s own platform on a first-come-first-served basis. They were informed that they would receive between ¥2 and ¥5 (equivalent to US $0.30-$0.80) for completing the questionnaire, according to their membership level. Before filling in the questionnaire, participants had to provide their informed written consent with signature confirmation. The informed consent page described the project’s background and purpose, the possible risks, the payment after completing the questionnaire, and the confidentiality of information and records. To be able to access the questionnaire, participants must have opened and scrolled through the informed consent description for at least 15 seconds and self-declared understanding the purpose and risks of the study before signing. The survey was administered between February 19 and March 28, 2021.

### Questionnaire

The questionnaire included 31 questions, partitioned into the following sections: informed consent and introduction, attitudes toward delivery and distribution of COVID-19 vaccines, and sociodemographic characteristics. The questionnaire was written in standard simplified Chinese and is shown in Multimedia Appendix 1. Participants had to answer a question to reach the next question.

### Data Quality Checks

First, we made sure that the KuRunData platform verified the time taken to complete the questionnaire to ensure that participants read questions before answering. Specifically, survey samples were deleted if the participant took less than 240 seconds or more than 900 seconds to complete the questionnaire. Second, questions included strings that tested for whether there was conflict in the respondents’ logic, for example, if the age selected by the respondent is too small to match the situation of marriage, education, and occupation selected by the respondent in any possible way. A total of 243 samples were deleted because of these reasons.

### Data Analysis

All analyses used sampling weights to account for the complex survey design. The sampling weights were the inverse of the probability of selecting participants given the following variables: sex, rural versus urban residence, and province. These probabilities were calculated using population counts from the 2020 China Statistical Yearbook within each province. In the second part of the questionnaire (on attitudes toward delivery and distribution of COVID-19 vaccines), questions with multiple options were combined as binary variables. For each question, we computed the percentage of participants who selected certain options to summarize the survey findings. For binomial proportions, we constructed 2-sided 95% CIs using the Wilson score interval. To examine how attitudes varied by participants’ characteristics, we regressed the binary response onto age (10-year age groups); sex; household income; educational attainment; rural versus urban residence; vocation; and whether a participant had a family member, friend, or acquaintance who they knew had been infected with COVID-19. All regressions were logistic regressions and included only one of these variables plus a binary indicator for each province (province-level fixed effects). We also performed multivariable regression to demonstrate the results with all these variables included in Multimedia Appendix 2, Table A2. In a separate analysis, we used ordinal probit regression as a robustness check and treated the response to Q4 and Q9 as ordinal variables, which takes on the values 1 to 4 for Q4 and 1 to 5 for Q9. For Q4, those with a rank of 1 have the highest willingness to provide COVID-19 vaccines to foreign countries, while those with a rank of 4 have the lowest. For Q9, those with a rank of 1 have the highest price, while those with a rank of 5 have the lowest. The related results are listed in Multimedia Appendix 2, Table A3.

### Ethics

The study was approved by the Research Ethics Commission of the Institute of Basic Medical Sciences, Chinese Academy of Medical Sciences (001-2021), and the Research Ethics Commission of Zhejiang University (003-2020).

## Results

### Sample Characteristics

A total of 10,000 participants were invited to take the survey. All respondents completed the whole survey. Selected participants’ sociodemographic characteristics are shown in Table 1, and the full table is listed in Multimedia Appendix 2, Table A1. A total of 4921 females and 5079 males completed the questionnaire. In the survey sample, 9% (900) of the participants were aged 18 or 19 years, the majority (n=7250, 72.5%) were aged 20-59 years, and 18.5% (n=1850) were 60 years or older. Only one-tenth (n=1063, 10.6%) of participants had never been to school or only been to elementary school. About one-third (n=3512, 35.1%) of participants had received high school or technical secondary school education, and one-third (n=3371, 33.7%) had completed an undergraduate degree. Most the of participants (n=9444, 94.4%) were of *Han* ethnicity. A majority of participants (n=5935, 59.4%) lived in urban areas. About 1.7% (n=171) of participants worked as a health care provider, including nurses (n=35, 0.4%), physicians (n=46, 0.5%), community health workers (n=51, 0.5%), pharmacists (n=13, 0.1%), and “other” health care providers (n=26, 0.3%).

**Table 1:** Sociodemographic characteristics of the survey participants.

Characteristic		Survey participants			Population of China^a^ (%)
		Proportion (weighted %)^b^	Participants, n (%)		
**Sex**					
	Female	48.9	4921 (49.2)	48.9	
**Age group (years)**					
	<20	10.1	900 (9.0)	21.9	
	20-29	17.2	1645 (16.5)	13.1	
	30-39	17.4	1895 (19.0)	15.7	
	40-49	20.3	1890 (18.9)	15.8	
	50-59	17.7	1820 (18.2)	15.3	
	≥60	17.3	1850 (18.5)	18.1	
**Rural-urban residency**					
	Urban	61.1	5935 (59.4)	60.6	
**Works as a health care provider**					
	No	98.3	9829 (98.3)	99.1	
	Nurse	0.4	35 (0.4)	0.3	
	Physician	0.5	46 (0.5)	0.3	
	Community health worker	0.4	51 (0.5)	<0.1	
	Pharmacist	0.1	13 (0.1)	<0.1	
	Other health care provider	0.3	26 (0.3)	0.2	

^a^As per the 2020 China Statistical Yearbook.

^b^Weighted using survey sampling weights.

### Attitude Toward Global Allocation of Chinese COVID-19 Vaccines

As shown in Table 2, about three-quarters of participants (75.6%, 95% CI 74.6%-76.5%) agreed to provide COVID-19 vaccines to foreign countries before fulfilling all domestic needs. Most participants (64.4%, 95% CI 63.3%-65.4%) preferred providing COVID-19 vaccines as a more appropriate way to aid foreign countries compared with offering financial support or sending medical teams. In terms of financial support, participants preferred providing assistance through international platforms (72.1%, 95% CI 71%-73.1%) rather than directly offering assistance to the foreign countries. If COVID-19 vaccines were provided to foreign countries, countries that have diplomatic relations with China were considered as the priority by 56.6% (95% CI 55.5%-57.7%) of participants. Considering the way to deliver vaccines to foreign countries, 77.3% (95% CI 76.3%-78.2%) of participants preferred to provide vaccine products (finished vaccine products or transferring vaccine technology) directly to relevant countries instead of providing vaccines via a delivery platform like COVAX. Less than one-quarter of participants (22.7%, 95% CI 21.8%-23.7%) agreed to provide COVID-19 vaccines to foreign countries at the same or even lower than the cost, and over one half (56.5%, 95% CI 55.4%-57.6%) kept the view that the Chinese government should bear a larger proportion of the cost.

**Table 2:** Summary of survey findings.

Survey question, combined response, and original response			Proportion, % (95% CI)
**In your opinion, if there is a shortage of Chinese COVID-19 vaccines, how should the domestic and international demand first be met?**			
	**Provide to foreign countries before satisfying all domestic needs**		
		The vaccination needs of global (both domestic and abroad) high-risk and high-danger groups should be met first before other needs are taken into consideration.	19.9 (19.0-20.8)
		The vaccination needs of domestic high-risk and high-danger groups should be met first before the vaccination needs abroad are supported.	55.7 (54.6-56.8)
	**Satisfy the vaccination needs of all Chinese people before providing to others**		
		The vaccination needs of all domestic groups should be met first before the vaccination needs abroad are supported.	22.7 (21.8-23.6)
		Only the domestic vaccination needs should be met, and the remaining vaccines should be taken as national strategic reserves without supporting the vaccination needs abroad.	1.8 (1.5-2.1)
**In response to the COVID-19 global pandemic, what do you think China should first consider in providing assistance to relevant countries?**			
	**To provide COVID-19 vaccines developed by Chinese scientific research institutions and enterprises**		
		Providing COVID-19 vaccines	64.3 (63.3-65.4)
	**To provide financial support, medical teams, or others**		
		Providing financial aid	13.5 (12.8-14.3)
		Send medical teams	18.6 (17.7-19.5)
		Others, please specify	1.0 (0.8-1.3)
		None of the above	2.5 (2.2-2.9)
**If COVID-19 vaccines that are developed by Chinese research institutions and companies are to support foreign countries, which countries do you think should be most supported?**			
	**Friendly countries that have diplomatic relations with China**		
		Countries with friendly diplomatic relations	56.7 (55.4-57.7)
	**Low-income countries, countries in need, or countries suggested by the WHO^a^ to support**		
		Lowest-income countries	6.0 (5.5-6.6)
		Any countries in need	21.0 (20.1-21.9)
		Countries recommended to be supported by the WHO	16.4 (15.6-17.2)
**In response to the COVID-19 global pandemic, what do you think China should first consider if it were to provide financial aid to other countries?**			
	**To provide financial support directly to foreign countries**		
		Donate the funds to the designated country, and the government of the recipient country will arrange its own pandemic prevention efforts	27.9 (26.9-29.0)
	**To provide financial support via authoritative international organizations or specialized organizations**		
		Donate the funds to international organizations (eg, the WHO) for comprehensive arrangement and coordination of the response to the pandemic	42.6 (41.5-43.8)
		Donate the funds to specialized vaccine organizations (eg, the GAVI^b^ Alliance) to purchase COVID-19 vaccines for less-developed countries	29.4 (28.4-30.5)
**What plan do you think China should prioritize if it were to provide the Chinese COVID-19 vaccines to other countries?**			
	**To provide finished vaccine products directly to foreign countries, or to transfer vaccine technology to relevant countries to allow local production**		
		Plan A: Directly providing vaccine products to relevant countries	61.2 (60.1-62.3)
		Plan B: Provide vaccine technology transfer to relevant countries and have their local enterprises produce the vaccines	16.1 (15.3-16.9)
	**To provide vaccines via the delivery platform of authoritative international organizations**		
		Plan C: Leverage the vaccine delivery platforms of international professional organizations	22.7 (21.8-23.7)
**In your opinion, at what price should the COVID-19 vaccines developed by the Chinese scientific institutions and enterprises be provided to foreign countries?**			
	**At market price or a small profit**		
		Market price	39.9 (38.8-41.0)
		A price with meager profits	37.4 (36.3-38.5)
	**At or even lower than cost price**		
		Cost price	18.0 (17.1-18.9)
		A price with meager loss	1.4 (1.2-1.7)
		Free of charge	3.3 (2.9-3.8)
**If the Chinese COVID-19 vaccines are priced below cost, who should bear the price loss when the vaccines are foreigners who are receiving foreign aid?**			
	**The government should bear a larger proportion of the loss than the enterprise.**		
		All borne by the enterprises	3.1 (2.8-3.5)
		Most borne by the enterprises and partially borne by the Chinese government	19.2 (18.3-20.1)
		Equally borne by the enterprises and the Chinese government	21.2 (20.3-22.1)
	**The enterprise should bear a larger proportion of the loss than the government.**		
		Most borne by the Chinese government and partially borne by the enterprises	36.9 (35.8-38.0)
		All borne by the Chinese government	19.7 (18.8-20.6)

^a^WHO: World Health Organization.

^b^GAVI: Global Alliance for Vaccines and Immunizations.

### Variation in Attitude Toward International Delivery of COVID-19 Vaccines by Sociodemographic Characteristics

Regarding the willingness to provide COVID-19 vaccines to foreign countries, participants who were female and living in a rural area tended to agree that COVID-19 vaccines can be provided to foreign countries before fulfilling all domestic needs (Table 3). Females were 1.18 (95% CI 1.07-1.32) times more likely than males to agree to provide COVID-19 vaccines to foreign countries. The probability that urban residents agreed was 0.89 (95% CI 0.79-0.99) that of rural residents. Multivariate regression and ordered logistic regression results showed the same results (see Multimedia Appendix 2, Tables A2 and A3).

In terms of COVID-19 vaccine pricing, urban residents and higher annual household income groups were more inclined to believe that COVID-19 vaccines should be provided abroad at market price, instead of at a lower price or free of charge (Table 3). The probability that urban residents agreed to provide COVID-19 vaccines abroad at a low price or free of charge was 0.88 (95% CI 0.78-0.98) that of rural residents. The probability that people whose annual household income was ¥90,000-¥119,999 (US $14,229-$18,972) agreed to provide COVID-19 vaccines abroad at a low price or free of charge was 0.62 (95% CI 0.49-0.80) that of the <¥ 30,000 (US $4743) income group. In multivariate regression, rural-urban residency was not significant, which might be explained by the collinearity between annual household income and rural-urban residency (see Multimedia Appendix 2, Table A2). The ordered logistic regression result showed the same results (see Multimedia Appendix 2, Table A3).

**Table 3:** Variation in attitude toward international delivery of COVID-19 vaccines by sociodemographic characteristics.^a^

Characteristic			Supporting COVID-19 vaccine provision to foreign countries before fulfilling all domestic needs^b^							Supporting COVID-19 vaccines as low-priced or free global public goods^c^				
			Participants, n (%)		OR^d^ (95% CI)		*P* value		Participants, n (%)			OR (95% CI)		*P* value
**Sex**														
	Male (n=5079)	3779 (74.4)		1 (ref)		N/A^e^		1110 (21.9)			1 (ref)		N/A	
	Female (n=4921)	3778 (76.8)		1.18 (1.07-1.32)		.002		1147 (23.3)			1.10 (0.98-1.22)		.10	
**Age group (years)**														
	18-19 (n=900)	724 (80.4)		1 (ref)		N/A		227 (25.2)			1 (ref)		N/A	
	20-29 (n=1645)	1299 (79.0)		0.84 (0.67-1.05)		.13		371 (22.6)			0.87 (0.70-1.08)		.21	
	30-39 (n=1895)	1400 (73.9)		0.67 (0.54-0.84)		<.001		441 (23.3)			0.88 (0.71-1.09)		.24	
	40-49 (n=1890)	1400 (74.1)		0.69 (0.55-0.85)		.001		437 (23.1)			0.89 (0.73-1.10)		.29	
	50-59 (n=1820)	1375 (75.5)		0.72 (0.58-0.90)		.004		383 (21.0)			0.75 (0.61-0.93)		.009	
	>60 (n=1850)	1359 (73.5)		0.64 (0.52-0.80)		<.001		398 (21.5)			0.80 (0.65-0.99)		.04	
**Annual household income (¥)**														
	<30,000 (n=572)	419 (73.3)		1 (ref)		N/A		173 (30.2)			1 (ref)		N/A	
	30,000-59,999 (n=1307)	1013 (77.5)		1.25 (0.97-1.61)		.09		306 (23.4)			0.72 (0.56-0.93)		.01	
	60,000-89,999 (n=1929)	1458 (75.6)		1.18 (0.93-1.51)		.18		459 (23.8)			0.72 (0.57-0.92)		.008	
	90,000-119,999 (n=1726)	1311 (76.0)		1.25 (0.98-1.60)		.07		386 (22.4)			0.62 (0.49-0.80)		<.001	
	120,000-149,999 (n=1726)	1317 (76.3)		1.25 (0.97-1.60)		.08		385 (22.3)			0.64 (0.50-0.82)		<.001	
	150,000-199,999 (n=1882)	1407 (74.8)		1.17 (0.91-1.49)		.22		364 (19.3)			0.56 (0.44-0.72)		<.001	
	≥200,000 (n=858)	632 (73.7)		1.08 (0.82-1.43)		.58		184 (21.4)			0.56 (0.42-0.75)		<.001	
**Education**														
	Never been to school (n=526)	398 (75.7)		1 (ref)		N/A		123 (23.4)			1 (ref)		N/A	
	Elementary school (n=537)	391 (72.8)		0.79 (0.57-1.10)		.16		119 (22.2)			1.10 (0.78-1.54)		.59	
	Middle school (n=1753)	1318 (75.2)		0.97 (0.74-1.27)		.83		384 (21.9)			1.04 (0.79-1.37)		.77	
	High school/technical secondary school (n=3512)	2679 (76.3)		1.02 (0.79-1.32)		.87		831 (23.7)			1.14 (0.88-1.47)		.32	
	College/undergraduate (n=3371)	2534 (75.2)		1.00 (0.78-1.29)		.99		731 (21.7)			0.96 (0.74-1.24)		.76	
	Graduate and above (n=301)	237 (78.7)		1.20 (0.80-1.81)		.37		69 (22.9)			1.03 (0.69-1.53)		.89	
**Rural-urban residency**														
	Rural (n=4065)	3123 (76.8)		1 (ref)		N/A		970 (23.9)			1 (ref)		N/A	
	Urban (n=5935)	4434 (74.7)		0.89 (0.79-0.99)		.03		1287 (21.7)			0.88 (0.78-0.98)		.02	
**Works as a health care provider**														
	No (n=9829)	7418 (75.5)		1 (ref)		N/A		2212 (22.5)			1 (ref)		N/A	
	Nurse (n=35)	30 (85.7)		2.44 (0.89-6.66)		.08		12 (34.3)			1.72 (0.79-3.73)		.17	
	Physician (n=46)	38 (82.6)		1.00 (0.43-2.36)		.99		10 (21.7)			1.09 (0.49-2.43)		.83	
	Community health worker (n=51)	40 (78.4)		1.06 (0.49-2.26)		.89		13 (25.5)			1.28 (0.63-2.61)		.49	
	Pharmacist (n=13)	10 (76.9)		1.13 (0.30-4.30)		.86		4 (30.8)			1.25 (0.34-4.56)		.74	
	Other health care provider (n=26)	21 (80.8)		1.40 (0.51-3.88)		.51		6 (23.1)			0.79 (0.30-2.12)		.64	
**Knows someone with a confirmed SARS-CoV-2 infection**														
	No (n=9972)	7537 (75.6)		1 (ref)		N/A		2253 (22.6)			1 (ref)		N/A	
	Self (n=3)	2 (66.7)		1.22 (0.11-14.11)		.87		0 (0.0)			0.00 (0.00-0.00)		<.001	
	Family member (n=3)	2 (66.7)		0.48 (0.04-5.57)		.56		0 (0.0)			0.00 (0.00-0.00)		<.001	
	Friend (n=8)	6 (75.0)		1.00 (0.19-5.23)		>.99		3 (37.5)			1.85 (0.39-8.73)		.44	
	Neighbor (n=3)	2 (66.7)		1.03 (0.08-13.07)		.98		0 (0.0)			0.00 (0.00-0.00)		<.001	
	Coworker (n=3)	1 (33.3)		0.05 (0.00-0.56)		.02		1 (33.3)			3.16 (0.29-34.90)		.35	
	Others (n=8)	7 (87.5)		8.43 (1.03-69.13)		.047		0 (0.0)			0.00 (0.00-0.00)		<.001	

^a^All regressions included only one of the variables (sex; age group; income; education; rural-urban residency; vocation; whether or not a participant has a family member, friend, or acquaintance who they know to have been infected with SARS-CoV-2) shown in the table and a binary indicator for each province (province-level fixed effects).

^b^“Satisfy the vaccination needs of all Chinese people before providing to others” is the reference response.

^c^“At market price or a small profit” is the reference response.

^d^OR: odds ratio.

^e^N/A: not applicable.

### Geographical Differences in People’s Attitude Toward International Delivery of COVID-19 Vaccines

**Figure 1:**
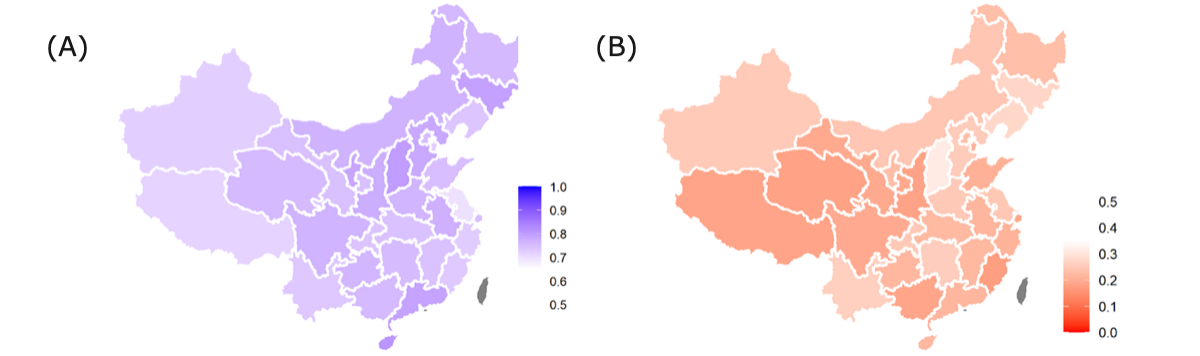
The proportion of the population by province (A) supporting COVID-19 vaccine provision to foreign countries before fulfilling all domestic needs and (B) supporting COVID-19 vaccines as low-priced or free global public goods.

There was a moderate degree of geographical variation in peoples’ attitudes toward international delivery of COVID-19 vaccines. The percentage of the population that supported provision of COVID-19 vaccines to foreign countries before fulfilling all domestic needs ranged from 69.7% (95% CI 64.8%-74.3%) in Jiangsu Province to 81% (95% CI 76.2%-85.1%) in Hainan Province (Figure 1a), and the percentage of the population that favored provision of COVID-19 vaccines at a low-price or as a free global public good ranged from 17.3% (95% CI 13.4%-22.1%) in Fujian Province to 31.2% (95% CI 26.2%-36.7%) in Shanxi Province (Figure 1b). As for Hubei Province, which was first exposed to serious COVID-19 prevalence in early 2020, the proportion of the above two questions were 74.4% (95% CI 69.1%-79%) and 22% (95% CI 17.7%-27%), respectively, and were among the middle of the geographical difference. There was a low degree of regional variation in people’s attitudes toward other questions on international delivery of COVID-19 vaccines, as detailed in Multimedia Appendix 2, Figure A1.

## Discussion

This study investigated population-level attitudes toward the global allocation of Chinese COVID-19 vaccines based on a large-scale online survey. Our study has four main findings. First, in general, the Chinese public strongly supports the provision of domestic vaccines as international assistance—even if herd immunity has not been achieved domestically. Second, participants had mixed preferences regarding multilateral and bilateral international assistance. In terms of financial support, most people preferred donation to international organizations such as the WHO rather than directly to specific countries; regarding COVID-19 vaccines on the other hand, most participants preferred direct bilateral provision of vaccines to relevant countries. Third, female participants and those living in a rural area tended to state a higher willingness to provide Chinese COVID-19 vaccines abroad. This may be because rural areas are vast, and participants from rural areas face a lower risk of COVID-19 than urban areas, but further research is necessary to determine a rationale. Fourth, participants with a high household income had a lower willingness to provide Chinese COVID-19 vaccines to foreign countries at low prices or free of charge compared to lower-income individuals.

Several factors may explain Chinese adults’ support for international vaccine assistance. First, China has thus far contained the epidemic through a series of nonpharmaceutical interventions such as prompt physical distancing [30,31]; an effective test, trace, and isolate system [32]; the adoption of facility-based isolation, widely used in Asia, but not in Western countries [33-36]; and clear communication and education [37,38]. As such, existing research has highlighted how Chinese residents often do not view themselves as “desperately needing” vaccination [39]. Second, the Chinese public receive messages from domestic key opinion leaders [40,41] as well as news from the national media and the WHO [42] that the virus causing COVID-19 will not disappear in the short term and is likely to circulate worldwide [43,44], and that the only way to end the global pandemic is through global collaboration to jointly contain the epidemic in all countries. Third, China has committed at the 2020 World Health Assembly to make Chinese vaccines a global public good and to ensure that they are provided to low- and middle-income countries at an affordable price [45]. This announcement that was widely reported via domestic channels [46-48] may have affected the attitudes of the Chinese public.

Participants in this study preferred providing funding via global institutions such as the WHO instead of direct bilateral economic aid during the pandemic, but in terms of COVID-19 vaccines, they preferred direct support to foreign countries. Scholars have used principal-agent theory to explain people’s preference toward multilateral or bilateral foreign aid and found that people often prefer multilateral foreign aid because they think it is a way to share responsibilities mutually with partner countries through the platform of international organizations. On the other hand, people often prefer bilateral aid because they think their country can have more control over the aid [11]. However, previous studies did not distinguish people’s attitudes on different aid categories such as direct economic aid or products. Based on these findings, we suggest that the Chinese public may recognize the benefit of providing financial support to the WHO because international health assistance requires collective action. However, the Chinese public may regard providing vaccines as a specific task, such that the advantage of delegation does not appear to outweigh the cost of foregoing the opportunity of choosing recipient countries.

Although people from different countries may have divergent views, the evidence from China suggests public support for international vaccine assistance. This finding has important policy implications for current global vaccine delivery and allocation work that could be particularly relevant for global policy makers within the WHO and in countries with considerable capability to produce vaccines. Although some policy approaches reflect a desire to complete domestic vaccination first before considering international assistance and exports [49], the evidence from China implies that the public may have different views—preferring instead to address international demand regardless of whether domestic herd immunity has been reached. Scientists and academics in other countries or anybody who is neutral in this matter could conduct national surveys to explore the public’s view. If the public supports the international assistance or export of COVID-19 vaccines, politicians may feel encouraged to stop stockpiling vaccines.

In addition, this research provides evidence for global policy makers such as the WHO to encourage and call on more countries to provide vaccine assistance in the name of public support. To date, the WHO and other global leaders working within COVAX have called on governments and the private sector to facilitate the global distribution and contribution of COVID-19 vaccines in an equitable way [50,51], but they have not yet appealed to the public. This research suggests that a majority of the Chinese public support international assistance with COVID-19 vaccination and that the public sees merit in financial support to the WHO during the pandemic. Regular global surveys on such preferences among the public of different nations could inform global leaders when making decisions about financial or in-kind support.

While a strength of this study is its nationwide coverage and relatively large sample size of 10,000 participants, it also has several important limitations. First, the survey was restricted to China, and thus, our findings may not be transferable to other countries or regions. Second, our sample of participants is unlikely to be representative of the general population in China because individuals had to be registered with KuRunData to be eligible for this study and were invited to participate on a first-come-first-served basis. Third, social desirability bias could have influenced participants to answer in ways that they thought they should, rather than how they truly felt.

In conclusion, participants in this online survey were generally supportive of the national government’s provision of domestically produced vaccines to other countries. However, preferences varied somewhat between population subgroups. Our findings could be useful for global and national policy makers as they seek to facilitate an equitable global allocation of COVID-19 vaccines.

## Appendix

Multimedia Appendix 1 Questionnaire text in English and Chinese.

Multimedia Appendix 2 Supplementary material.

## Abbreviations

GAVI: Global Alliance for Vaccines and Immunizations
WHO: World Health Organization
