# Supplementary material for: Attitudes Toward the Global Allocation of Chinese COVID-19 Vaccines: Cross-sectional Online Survey of Adults Living in China
Source: JMIR Public Health Surveill. 2022 Jun 7;8(6):e33484. doi: 10.2196/33484 (PMC9177168; doi:10.2196/33484)
Supplement: Multimedia Appendix 1 [file publichealth_v8i6e33484_app1.docx]

**Multimedia Appendix 1. Questionnaire text in English and Chinese.**

|  | Informed Consent Form  The Chinese Academy of Medical Sciences, Peking Union Medical College and Kurundata are conducting an ongoing web-based survey on “Public Attitudes towards the COVID-19 Vaccine Delivery, Pricing and Eligibility: A Representative Study of 10,000 Respondents across China”. Your participation can provide data needed for the study. Please read and understand the study in detail and feel free to ask any questions you may have. If you agree to participate in this project, please confirm that you meet the eligibility criteria in order to complete the informed consent and check “I agree to participate in this survey”.  I. Purpose of Research: The objective of this research project is to study the attitudes of the Chinese population towards the priority delivery of Chinese COVID-19 vaccines. The results of this study will provide empirical data for the development of relevant response strategies. It is hoped that the project will provide key information about science and public health policy as well as supporting evidence for the government’s foreign aid decisions, vaccine delivery, pricing and public vaccination.  II. Research methods: A certain number of participants over 18 years of age who meet the eligibility criteria will be recruited for this study on a voluntary basis. This study is a questionnaire survey to collect the attitudes of the Chinese population towards the priority delivery of Chinese COVID-19 vaccines. The research period is approximately 12 months.  III. Rights and obligations: Your participation in this study is entirely voluntary. You may choose not to participate in the study, and you may request to withdraw at any time without any reason and your rights will not be affected in any way. If you agree to participate in this study, we will conduct a survey on you to learn about your general situation, knowledge and awareness of protection measures.  IV. Expected risks and measures: Respondents can stop participating in the research at any time if they feel uncomfortable with some of the questions in the questionnaire.  V. Expected social benefits: The findings of this survey will provide information on the COVID-19 vaccine delivery, pricing, vaccination and the development of related policies.  VI. Individual benefits: To express our gratitude to you for participating in the survey, you will receive the corresponding bonus points after completing the online survey.  VII. Confidentiality covenant: The information you fill in is for the reference of the researchers only. When the information or findings are applied, no personal information of yours will be disclosed.  VIII. Contact information: If you have any questions related to this study, please feel free to contact us via yangjt@pumc.edu.cn.  IX. Signing of Informed Consent: If you check the “I agree to participate in this survey” box, it will mean that you have correctly understood the study as a research subject and are willing to fill in the questionnaire on a voluntary basis. |
| --- | --- |
|  | 1. I agree to participate in this survey |
|  | 2. I don’t agree to participate in this survey |

| 33 | At present, the COVID-19 is still rampaging around the world, and the COVID-19 vaccines are considered to be a powerful weapon with the potential to end the global pandemic. Currently, the COVID-19 vaccines developed by the Chinese scientific institutions and enterprises (a.k.a “Chinese COVID-19 vaccines”) have been launched towards the market and approved for emergency use in China and some foreign countries.  Therefore, we would like to know your attitudes and thoughts on the Chinese COVID-19 vaccine delivery and distribution. It will take you about 5 to 8 minutes to complete the questionnaire. Your personal information will be completely anonymous. Thank you for your support and cooperation! |
| --- | --- |

| Q1 | How likely do you think it is that you will be notified for vaccination against COVID-19 within 2021? |
| --- | --- |
|  | 1. Completely unlikely |
|  | 2. Unlikely |
|  | 3. Likely |
|  | 4. Completely likely |
|  | 5. Has been vaccinated |

| Q2 | What’s your attitude towards the following issues? | | | | | |
| --- | --- | --- | --- | --- | --- | --- |
|  |  | Strongly disagree | Disagree | Neutral | Agree | Strongly agree |
|  | 1. If the Chinese COVID-19 vaccine receives approval for the market launch from the National Medical Products Administration, I hope that my family and I can be vaccinated as soon as possible. (Notes: The National Medical Products Administration is responsible for vaccine quality regulation in China.) |  |  |  |  |  |
|  | 1. If the Chinese COVID-19 vaccine is technically certified by the World Health Organization, I hope that my family and I can be vaccinated as soon as possible. (Notes: The World Health Organization is responsible for technical certification of vaccine procurement in the United Nations system.) |  |  |  |  |  |
|  | 1. If the Chinese COVID-19 vaccine is included into the Global Procurement List of The GAVI Alliance, I hope that my family and I can be vaccinated as soon as possible. (Notes: The GAVI Alliance has provided various vaccines for 49% of the children in the world, and it's the world’s largest vaccine procurement platform.) |  |  |  |  |  |

| Q3 | China plans to vaccinate three groups of people against COVID-19 successively.   \| Group I \| High-risk Group \| This group mainly contains frontline medical and pandemic prevention workers, border and port workers, and those who are obligated to go to areas or countries with high risk of infection due to work-related reasons, not to mention those who ensure the basic operation of the cities. \| \| --- \| --- \| --- \| \| Group II \| High-danger Group \| This group mainly contains seniors, children, pregnant women and people with underlying medical conditions who will have a far greater rate of severe or critically ill cases if infected with the COVID-19 compared to other groups. \| \| Group III \| General Group \| Other people. \|   Do you agree with the current delivery plan? |
| --- | --- | --- | --- | --- | --- | --- | --- | --- | --- | --- |
|  | 1. Yes |
|  | 2. No |

| Q3A | If no, which group do you think should be given priority for vaccination? |
| --- | --- |

| Q4 | In your opinion, if there is a shortage of Chinese COVID-19 vaccines, how should the domestic and international demand first be met? |
| --- | --- |
|  | 1. The vaccination needs of global (both domestic and abroad) high-risk and high-danger groups should be met first before other needs are taken into consideration. |
|  | 1. The vaccination needs of domestic high-risk and high-danger groups should be met first before the vaccination needs abroad are supported. |
|  | 1. The vaccination needs of all domestic groups should be met first before the vaccination needs abroad are supported. |
|  | 1. Only the domestic vaccination needs should be met and the remaining vaccines should be taken as national strategic reserves without supporting the vaccination needs abroad. |

| Q5 | In response to the COVID-19 global pandemic, what way do you think China should first consider in providing assistance to relevant countries? |
| --- | --- |
|  | 1. Providing financial aid |
|  | 2. Providing COVID-19 vaccines |
|  | 3. Send medical teams |
|  | 4. Others, please specify: |
|  | 5. None of the above |

| Q6 | If COVID-19 vaccines that are developed by Chinese research institutions and companies are to support foreign countries, which countries do you think should be most supported? |
| --- | --- |
|  | 1. Countries with friendly diplomatic relations |
|  | 2. Lowest-income countries |
|  | 3. Any countries in need |
|  | 4. Countries recommended to be supported by the WHO |

| Q7 | In response to the COVID-19 global pandemic, what way do you think China should first consider if it were to provide financial aid to other countries? |
| --- | --- |
|  | 1. Donate the funds to the designated country, and the government of the recipient country will arrange its own pandemic prevention efforts. |
|  | 1. Donate the funds to international organizations (such as the World Health Organization) for comprehensive arrangement and coordination of the response to the pandemic. |
|  | 1. Donate the funds to specialized vaccine organizations (such as the GAVI Alliance) to purchase COVID-19 vaccines for the less-developed countries. |

| Q8 | What plan do you think China should prioritize if it were to provide the Chinese COVID-19 vaccines to other countries?   \|  \| Strengths \| Weakness \| \| --- \| --- \| --- \| \| Plan A: Directly providing vaccine products to relevant countries \| China can decide its own distribution scheme. \| Due to vaccine logistics and cold chain technology restrictions, there will be a limited number of vaccines to support foreign countries in the short term. \| \| Plan B: Provide vaccine technology transfer to relevant countries and have their local enterprises produce the vaccines \| The local enterprises produce the vaccines. \| There will be no production limits, but many less-developed countries do not have the capacity to produce vaccines. \| \| Plan C: Leverage the vaccine delivery platforms of international professional organizations. \| The cold chain technology problem will be resolved. \| Lower China’s autonomy in deciding which countries to provide vaccines to. \| |
| --- | --- | --- | --- | --- | --- | --- | --- | --- | --- | --- | --- | --- | --- |
|  | 1. Plan A |
|  | 2. Plan B |
|  | 3. Plan C |

| Q9 | In your opinion, at what price should the COVID-19 vaccines developed by the Chinese scientific institutions and enterprises be provided to foreign countries? |
| --- | --- |
|  | 1. Market price |
|  | 2. A price with meager profits |
|  | 3. Cost price |
|  | 4. A price with meager loss |
|  | 5. Free of charge |

| Q10 | If the Chinese COVID-19 vaccines are priced below cost, who should bear the price loss when the vaccinees are foreigners who are receiving foreign aid? |
| --- | --- |
|  | 1. All borne by the enterprises |
|  | 2. Most borne by the enterprises and partially borne by the Chinese government |
|  | 3. Equally borne by the enterprises and the Chinese government |
|  | 4. Most borne by the Chinese government and partially borne by the enterprises |
|  | 5. All borne by the Chinese government |

| Q11 | Do you agree that our country should spend more on aid to other countries in the future, even if it means spending less domestically? |
| --- | --- |
|  | 1. Strongly agree |
|  | 2. Agree |
|  | 3. Not sure about it |
|  | 4. Disagree |
|  | 5. Strongly disagree |

| Q12 | If the total amount of aid remains the same, which of the following areas would you like China to spend more of its aid funds on in the future? |
| --- | --- |
|  | 1. Directly provide the aid to recipient countries |
|  | 2. Donating the funds to international organizations (such as the United Nations) which will distribute them to other countries |

| Q13 | If 100 people are going to be infected with the COVID-19, how many of them do you think can be prevented from infection by early vaccination? |
| --- | --- |
|  | 1. 100 |
|  | 2. 90-99 |
|  | 3. 80-89 |
|  | 4. 70-79 |
|  | 5. <70 |

| Q14 | If 100 people are going to die of the COVID-19, how many of them do you think can be prevented from death by early vaccination? |
| --- | --- |
|  | 1. 100 |
|  | 2. 90-99 |
|  | 3. 80-89 |
|  | 4. 70-79 |
|  | 5. < 70 |

| Q15 | If 100,000 people are vaccinated against the COVID-19, how many people on average do you think will need to be hospitalized due to severe adverse reactions? |
| --- | --- |
|  | 1. 0 |
|  | 2. 1-5 |
|  | 3. 6-10 |
|  | 4. 11-15 |
|  | 5. >15 |

| Q16 | If 100,000 people are vaccinated against the COVID-19, how many people on average do you think will die of severe adverse reactions? |
| --- | --- |
|  | 1. 0 |
|  | 2. 1-5 |
|  | 3. 6-10 |
|  | 4. 11-15 |
|  | 5. >15 |

| H1 | Have you ever been diagnosed with COVID-19? |
| --- | --- |
|  | 1. Yes |
|  | 2. No |

| H2 | Has any of your family members, neighbors, colleagues, friends or other people you know been diagnosed with COVID-19? |
| --- | --- |
|  | 1. Family members |
|  | 2. Friends |
|  | 3. Neighbors |
|  | 4. Colleagues |
|  | 5. Other people I know, please specify: _______ |

| H3 | During the COVID-19 outbreak, have you ever been quarantined as a close contact or suspected patient? |
| --- | --- |
|  | 1. Yes |
|  | 2. No |

| H4 | What is your age? Please fill in a figure. No decimals. |
| --- | --- |
|  | 1. |

| H4A | age |
| --- | --- |
|  | 1. 18-19yrs |
|  | 2. 20-29yrs |
|  | 3. 30-39yrs |
|  | 4. 40-49yrs |
|  | 5. 50-59yrs |
|  | 6. >60yrs |

| H5 | Your sex is: |
| --- | --- |
|  | 1. Male |
|  | 2. Female |

| H6 | In which province do you live now? |
| --- | --- |
|  | 1. Hebei Province |
|  | 2. Shanxi Province |
|  | 3. Liaoning Province |
|  | 4. Jilin Province |
|  | 5. Heilongjiang Province |
|  | 6. Jiangsu Province |
|  | 7. Zhejiang Province |
|  | 8. Anhui Province |
|  | 9. Fujian Province |
|  | 10. Jiangxi Province |
|  | 11. Shandong Province |
|  | 12. Henan Province |
|  | 13. Hubei Province |
|  | 14. Hunan Province |
|  | 15. Guangdong Province |
|  | 16. Hainan Province |
|  | 17. Sichuan Province |
|  | 18. Guizhou Province |
|  | 19. Yunnan Province |
|  | 20. Shaanxi Province |
|  | 21. Gansu Province |
|  | 22. Qinghai Province |
|  | 23. Inner Mongolia Autonomous Region |
|  | 24. Guangxi Autonomous Region |
|  | 25. Tibet Autonomous Region |
|  | 26. Ningxia Hui Autonomous Region |
|  | 27. Xinjiang Uygur Autonomous Region |
|  | 28. Beijing |
|  | 29. Shanghai |
|  | 30. Tianjin |
|  | 31. Chongqing |

| H8 | Do you live in a rural area or an urban area? |
| --- | --- |
|  | 1. A rural area |
|  | 2. An urban area |

| H9 | In which province did you spend your 2020 Spring Festival? |
| --- | --- |
|  | 1. Hebei Province |
|  | 2. Shanxi Province |
|  | 3. Liaoning Province |
|  | 4. Jilin Province |
|  | 5. Heilongjiang Province |
|  | 6. Jiangsu Province |
|  | 7. Zhejiang Province |
|  | 8. Anhui Province |
|  | 9. Fujian Province |
|  | 10. Jiangxi Province |
|  | 11. Shandong Province |
|  | 12. Henan Province |
|  | 13. Hubei Province |
|  | 14. Hunan Province |
|  | 15. Guangdong Province |
|  | 16. Hainan Province |
|  | 17. Sichuan Province |
|  | 18. Guizhou Province |
|  | 19. Yunnan Province |
|  | 20. Shaanxi Province |
|  | 21. Gansu Province |
|  | 22. Qinghai Province |
|  | 23. Inner Mongolia Autonomous Region |
|  | 24. Guangxi Autonomous Region |
|  | 25. Tibet Autonomous Region |
|  | 26. Ningxia Hui Autonomous Region |
|  | 27. Xinjiang Uygur Autonomous Region |
|  | 28. Beijing |
|  | 29. Shanghai |
|  | 30. Tianjin |
|  | 31. Chongqing |

| H11 | Was the place where you spent the 2020 Spring Festival a rural area or an urban area ? |
| --- | --- |
|  | 1. A rural area |
|  | 2. An urban area |

| H12 | What is the highest level of education you have completed? (If you are still in school, please select the highest education you have completed.) |
| --- | --- |
|  | 1. Never been to school |
|  | 2. Primary school |
|  | 3. Junior high school |
|  | 4. Senior high school/technical secondary school |
|  | 5. Junior college/Undergraduate |
|  | 6. Postgraduate and above |

| H13 | Which of the following industries do you currently work in? |
| --- | --- |
|  | 1. Agriculture, forestry, animal husbandry and fishery |
|  | 2. Mining |
|  | 3. Manufacturing |
|  | 4. Electricity, gas and water production and supply |
|  | 5. Construction |
|  | 6. Transportation, warehousing and postal services |
|  | 7. Information transmission, computer services and software |
|  | 8. Wholesale and retail |
|  | 9. Hotel and food services |
|  | 10. Financial services |
|  | 11. Real estate |
|  | 12. Leasing and business services |
|  | 13. Scientific research, technical services and geological exploration |
|  | 14. Hydraulic engineering, environment and public facilities management |
|  | 15. Residential service and other services |
|  | 16. Education |
|  | 17. Health, social security and welfare |
|  | 18. Culture, sports and entertainment |
|  | 19. Public management and social organization |
|  | 20. International organization |
|  | 21. Others |

| H14 | Are you in the healthcare sector? Are you a nurse, a doctor, a community health worker or a pharmacist? |
| --- | --- |
|  | 1. No, I am not in the healthcare industry |
|  | 2. A nurse |
|  | 3. A doctor |
|  | 4. A community health worker |
|  | 5. A pharmacist |
|  | 6. Other workers in the healthcare industry, please specify: |

| H15 | What’s your nationality? Please select the appropriate option. |
| --- | --- |
|  | 1. Han |
|  | 2. Hui |
|  | 3. Tibetan |
|  | 4. Zhuang |
|  | 5. Manchu |
|  | 6. Others, please specify: |

| H16 | What is the total annual income (RMB) of your family? |
| --- | --- |
|  | 1. <30000 |
|  | 2. 30000-60000 |
|  | 3. 60000-90000 |
|  | 4. 90000-120000 |
|  | 5. 120000-150000 |
|  | 6. 150000-200000 |
|  | 7. 200000+ |

| H17 | Please choose according to the level of agreement with the following statements. The number from 1 to 5 indicates an increasing degree of agreement. | | | | | |
| --- | --- | --- | --- | --- | --- | --- |
|  |  | Strongly disagree | Disagree | Neutral | Agree | Strongly agree |
|  | 1. If I was infected with the COVID-19, my health would be seriously compromised. |  |  |  |  |  |
|  | 2. I think the COVID-19 is more serious than the flu. |  |  |  |  |  |
|  | 3. Even if I had another disease, I would not go to the hospital because I would be exposed to COVID-19 infection in the hospital. |  |  |  |  |  |
|  | 4. The COVID-19 will cause serious damage to my community. |  |  |  |  |  |
|  | 5. I am more likely to be infected with the COVID-19 than others. |  |  |  |  |  |
|  | 6. I believe that I can resist the COVID-19. |  |  |  |  |  |
|  | 7. I believe I can protect myself from the COVID-19 better than anyone else. |  |  |  |  |  |
|  | 8. I am afraid of the COVID-19. |  |  |  |  |  |
|  | 9. The COVID-19 has severely hurt the economy. |  |  |  |  |  |

**Text A1. Questionnaire text in Chinese version**

| Q32 | **知情同意书**        中国医学科学院 北京协和医学院与Kurundata库润数据公司正在进行的基于网络开展“调查民众对新冠疫苗交付、定价、资格认证态度的建议：对中国全国10000名受访者的代表性研究”，您的参与能为研究提供需要的数据。请您详细阅读并理解该项研究，如有任何疑问，请随时提出。若您同意参加此项目,可以在确认符合入选条件的情况下，勾选“我同意参加本次调查”，完成知情同意。       一、**研究目的**：本研究项目的目标旨在研究中国民众对中国新冠疫苗优先交付的态度。研究成果将为制定相关应对策略提供实证数据。希望通过项目的开展，提供有关科学和公共卫生政策的关键信息，为政府对外援助决策、疫苗交付、定价及公众疫苗接种提供支撑性证据。       二、**研究方法**：本研究招募一定数量的年满十八岁以上，符合入选条件且自愿参加者招募至本研究。本研究为问卷调查，通过问卷调查，收集中国民众对中国新冠疫苗优先交付的态度，研究周期约12个月。       三、**权利与义务**：您参加本项研究完全是自愿的。您可以选择不参加本项研究，也可以在任何时候不需要任何理由要求退出，您的权益不会因此受到任何影响。如果您同意参加本研究，我们将对您进行调研，了解您的一般情况、防护相关知识及认知等情况。       四、**预期风险及措施**：当受访者对部分问卷问题感到不适时，可以随时停止参与调研。       五、**预期社会收益**：本次调查结果将为新冠肺炎疫苗交付、定价、接种以及制定相关政策提供信息。       六、**个人收益**：为了表达对您参与问卷调查的感谢，您在完成线上问卷后，将收到对应积分奖励。       七、**保密约定**：您所填信息仅供研究人员查阅。应用信息或结果时，并不会涉及您的任何个人隐私信息。       八、**联系方式**：如果您有与本研究相关的任何问题，请随时联系我们：yangjt@pumc.edu.cn       九、**知情同意书签署**：勾选“我同意参加本次调查”者，即表示您作为调研对象已正确理解该研究，并自愿填写调查问卷。**[SR]** |
| --- | --- |
|  | 1、我同意参加本次调查 |
|  | 2、我不同意参加本次调查 |

| 33 | 当前，新冠病毒仍然在全球范围内肆虐，新冠疫苗被认为是有可能终结此次新冠疫情全球大流行的有力武器。目前，由中国科研机构和企业研发的新冠疫苗（以下简称中国新冠疫苗）已经在国内和国外一些国家上市或者获批紧急使用。     为此，我们希望了解您在新冠疫苗交付和分配方面的态度和想法。完成本次问卷大约需要花费您5-8分钟时间。我们将对您的个人信息采取完全匿名处理，感谢您的支持与配合！**[M]** |
| --- | --- |

| Q1 | 您认为在2021年内，您被通知可以接种新冠疫苗的可能性有多大？**[SR]** |
| --- | --- |
|  | 1、完全没有可能 |
|  | 2、没有可能 |
|  | 3、有可能 |
|  | 4、完全有可能 |
|  | 5、已经接种 |

| Q2 | 您对以下问题的态度是？**[MA]** | | | | | |
| --- | --- | --- | --- | --- | --- | --- |
|  |  | 完全同意 | 同意 | 中立 | 不同意 | 完全不同意 |
|  | 1、如果中国新冠疫苗得到了**中国国家药品监督管理局的上市批准**，我希望自己和家人尽快接种疫苗。(注释：中国国家药品监督管理局负责我国疫苗的质量监管工作)。 |  |  |  |  |  |
|  | 2、如果有中国 新冠疫苗获得**世界卫生组织的技术认证**，我希望自己和家人尽快接种。（注释：世界卫生组织负责联合国系统疫苗采购的技术认证工作）。 |  |  |  |  |  |
|  | 3、如果有中国的 新冠疫苗进入**全球疫苗免疫联盟的全球采购名单**，我希望自己和家人尽快接种。（注释：全球疫苗免疫联盟为全球49%的儿童提供过各类疫苗接种，是全世界最大的疫苗采购平台）。 |  |  |  |  |  |

| Q3 | 我国计划将按照以下三类人群，依此接种新冠疫苗。   \| 第一类 \| 高风险人群 \| 主要是指一线的医疗防疫人员，边境、口岸的工作人员，还有由于工作原因必须要去高感染风险地区或者国家的工作人员，以及保证城市基本运行的一些工作人员。 \| \| --- \| --- \| --- \| \| 第二类 \| 高危人群 \| 主要是指老人、儿童、孕妇以及患有基础疾病的人群。这类人群一旦感染新冠，出现重症或者危重症的比例远远大于其他人群 \| \| 第三类 \| 普通人群 \| 其他人。 \|   您是否认同现有的交付方案？ **[SR]** |
| --- | --- | --- | --- | --- | --- | --- | --- | --- | --- | --- |
|  | 1、是 |
|  | 2、 否 |

| Q3A | 如果不支持，您认为需要优先考虑接种的具体人群是？**[T]** |
| --- | --- |

| Q4 | 您认为中国科研机构和企业研发的新冠疫苗，如果出现供应短缺，应该如何优先满足国内外的需求？**[SR]** |
| --- | --- |
|  | 1、 先满足全球（包括国内和国外）高风险和高危群体的疫苗接种需求，再考虑其他需求。 |
|  | 2、先满足国内高风险和高危群体的疫苗接种需求，再支援国外需求。 |
|  | 3、 先满足所有国内民众的疫苗接种需求，再支援国外需求。 |
|  | 4、只满足国内需求，剩余的疫苗作为国家战略储备，不支援国外需求。 |

| Q5 | 在应对全球新冠疫情中，您认为中国应该优先考虑以何种方式对相关国家提供援助？**[SR]** |
| --- | --- |
|  | 1、提供资金援助 |
|  | 2、提供新冠疫苗 |
|  | 3、派遣医疗队 |
|  | 4、其他，请说明 |
|  | 5、都不考虑 |

| Q6 | 如果中国科研机构和企业研发的新冠疫苗支援国外，您认为最应该支援什么样的国家？**[SR]** |
| --- | --- |
|  | 1、 友好邦交国家 |
|  | 2、 最低收入国家 |
|  | 3、 任何有需要的国家 |
|  | 4、世界卫生组织建议支援的国家 |

| Q7 | 在应对全球新冠疫情中，如果中国政府要向其他国家提供资金援助，您认为应该优先考虑何种形式？**[SR]** |
| --- | --- |
|  | 1、将钱捐给指定国家，由受助国政府自行安排抗疫工作 |
|  | 2、 将钱捐给国际组织（例如世界卫生组织），由其全面统筹和协调各方面抗疫工作 |
|  | 3、将钱捐给疫苗专业组织（例如全球疫苗免疫联盟），为欠发达国家地区采购新冠疫苗 |

| Q8 | 您认为如果中国向国外提供新冠疫苗，应该优先通过何种途径？   \|  \| 优势 \| 劣势 \| \| --- \| --- \| --- \| \| 方案A: 向相关国家直接提供疫苗成品 \| 我国能够自主决定分配方案 \| 疫苗物流冷链技术等限制，短期内支援国外的疫苗数量比较有限 \| \| 方案B: 向相关国家提供疫苗技术转让，由当地企业进行疫苗生产。 \| 由当地企业进行疫苗生产 \| 这样做没有量产限制，但很多欠发达国家没有疫苗生产能力 \| \| 方案C：借助国际专业组织的疫苗交付平台。 \| 能够解决冷链技术等问题 \| 降低我国自主决定向哪些国家提供疫苗的灵活性 \|   **[SR]** |
| --- | --- | --- | --- | --- | --- | --- | --- | --- | --- | --- | --- | --- | --- |
|  | 1、方案A |
|  | 2、方案B |
|  | 3、方案C |

| Q9 | 您认为由中国科研机构和企业研发的新冠疫苗，应该以什么样的价格水平提供给国外？**[SR]** |
| --- | --- |
|  | 1、市场价 |
|  | 2、微薄盈利 |
|  | 3、成本价 |
|  | 4、微薄亏损 |
|  | 5、免费 |

| Q10 | 当接种对象是对外支援的国外民众时，如果中国的新冠疫苗定价低于成本，亏损的部分应该由谁来承担？**[SR]** |
| --- | --- |
|  | 1、全部由企业承担 |
|  | 2、大部分由企业承担，小部分由中国政府承担 |
|  | 3、企业和中国政府平摊 |
|  | 4、大部分由中国政府承担，小部分由企业承担 |
|  | 5、全部由中国政府承担 |

| Q11 | 您是否同意我国未来应该投入更多经费去援助其他国家，哪怕意味着减少国内开支？**[SR]** |
| --- | --- |
|  | 1、非常同意 |
|  | 2、同意 |
|  | 3、不确定 |
|  | 4、不同意 |
|  | 5、非常不同意 |

| Q12 | 如果援助资金总量不变，您希望中国未来将援助资金更多用于以下哪个方面？**[SR]** |
| --- | --- |
|  | 1、向具体国家直接提供援助 |
|  | 2、把钱交给国际组织，再由国际组织（例如联合国）分配给其他国家 |

| Q13 | 如果有100个人将被新冠病毒感染，您认为提前接种新冠疫苗能够防止其中多少人感染新冠病毒？**[SR]** |
| --- | --- |
|  | 1、100 |
|  | 2、90-99 |
|  | 3、80-89 |
|  | 4、70-79 |
|  | 5、<70 |

| Q14 | 如果有100人将死于新冠病毒感染，您认为提前接种新冠疫苗能够防止其中多少人因为新冠病毒感染而死亡？**[SR]** |
| --- | --- |
|  | 1、100 |
|  | 2、90-99 |
|  | 3、80-89 |
|  | 4、70-79 |
|  | 5、< 70 |

| Q15 | 如果有十万人接种新冠疫苗，您认为平均将有多少人会因为出现严重接种不良反应而需要住院治疗？**[SR]** |
| --- | --- |
|  | 1、 0 |
|  | 2、1-5 |
|  | 3、 6-10 |
|  | 4、11-15 |
|  | 5、>15 |

| Q16 | 如果有十万人接种新冠疫苗，您认为平均将有多少人会因为出现严重不良接种反应而死亡？**[SR]** |
| --- | --- |
|  | 1、0 |
|  | 2、1-5 |
|  | 3、6-10 |
|  | 4、11-15 |
|  | 5、>15 |

| H1 | 您是否曾被诊断出患有新冠肺炎？**[SR]** |
| --- | --- |
|  | 1、是 |
|  | 2、否 |

| H2 | 您的家庭成员、邻居、同事、朋友或您认识的其他人中是否有人被诊断出患有新冠肺炎？**[SR]** |
| --- | --- |
|  | 1、家庭成员 |
|  | 2、朋友 |
|  | 3、邻居 |
|  | 4、同事 |
|  | 5、我认识的其他人，请说明：_______ |

| H3 | 在新冠疫情期间，您是否有作为新冠患者密切接触者或者疑似患者而被集中隔离的经历？**[SR]** |
| --- | --- |
|  | 1、是 |
|  | 2、否 |

| H4 | 您的年龄是？请填写数字，不允许填写小数。**[MT]** |
| --- | --- |
|  | 1、 |

| H4A | age**[DU]** |
| --- | --- |
|  | 1、18-19yrs |
|  | 2、20-29yrs |
|  | 3、30-39yrs |
|  | 4、40-49yrs |
|  | 5、50-59yrs |
|  | 6、>60yrs |

| H5 | 您的性别是**[SR]** |
| --- | --- |
|  | 1、男 |
|  | 2、女 |

| H6 | 你现在居住在哪个省份？**[SR]** |
| --- | --- |
|  | 1、河北省 |
|  | 2、山西省 |
|  | 3、辽宁省 |
|  | 4、吉林省 |
|  | 5、黑龙江省 |
|  | 6、江苏省 |
|  | 7、浙江省 |
|  | 8、安徽省 |
|  | 9、福建省 |
|  | 10、江西省 |
|  | 11、山东省 |
|  | 12、河南省 |
|  | 13、湖北省 |
|  | 14、湖南省 |
|  | 15、广东省 |
|  | 16、海南省 |
|  | 17、四川省 |
|  | 18、贵州省 |
|  | 19、云南省 |
|  | 20、陕西省 |
|  | 21、甘肃省 |
|  | 22、青海省 |
|  | 23、内蒙古自治区 |
|  | 24、广西壮族自治区 |
|  | 25、西藏自治区 |
|  | 26、宁夏回族自治区 |
|  | 27、新疆维吾尔自治区 |
|  | 28、北京市 |
|  | 29、上海市 |
|  | 30、天津市 |
|  | 31、重庆市 |

| H8 | 请问您居住的是农村还是城镇？**[SR]** |
| --- | --- |
|  | 1、农村 |
|  | 2、城镇 |

| H9 | 您在2020年春节居住在哪个省份？**[SR]** |
| --- | --- |
|  | 1、河北省 |
|  | 2、山西省 |
|  | 3、辽宁省 |
|  | 4、吉林省 |
|  | 5、黑龙江省 |
|  | 6、江苏省 |
|  | 7、浙江省 |
|  | 8、安徽省 |
|  | 9、福建省 |
|  | 10、江西省 |
|  | 11、山东省 |
|  | 12、河南省 |
|  | 13、湖北省 |
|  | 14、湖南省 |
|  | 15、广东省 |
|  | 16、海南省 |
|  | 17、四川省 |
|  | 18、贵州省 |
|  | 19、云南省 |
|  | 20、陕西省 |
|  | 21、甘肃省 |
|  | 22、青海省 |
|  | 23、内蒙古自治区 |
|  | 24、广西壮族自治区 |
|  | 25、西藏自治区 |
|  | 26、宁夏回族自治区 |
|  | 27、新疆维吾尔自治区 |
|  | 28、北京市 |
|  | 29、上海市 |
|  | 30、天津市 |
|  | 31、重庆市 |

| H11 | 请问2020年春节您居住的所在地是农村还是城镇?**[SR]** |
| --- | --- |
|  | 1、农村 |
|  | 2、城镇 |

| H12 | 您的最高学历是？（如果现在依然在校读书，请选择您已念完的最高学历）**[SR]** |
| --- | --- |
|  | 1、没上过学 |
|  | 2、小学 |
|  | 3、初中 |
|  | 4、高中/中专 |
|  | 5、大专/本科 |
|  | 6、研究生及以上 |

| H13 | 您目前所从事的工作属于以下哪个行业？**[SR]** |
| --- | --- |
|  | 1、农、林、牧、渔业 |
|  | 2、采矿业 |
|  | 3、制造业 |
|  | 4、电力、煤气及水的生产和供应业 |
|  | 5、建筑业 |
|  | 6、交通运输、仓储及邮政业 |
|  | 7、信息传输、计算机服务和软件业 |
|  | 8、批发和零售业 |
|  | 9、住宿和餐饮业 |
|  | 10、金融业 |
|  | 11、房地产业 |
|  | 12、租赁和商务服务业 |
|  | 13、科学研究、技术服务和地质勘查业 |
|  | 14、水利、环境和公共设施管理业 |
|  | 15、居民服务和其他服务业 |
|  | 16、教育业 |
|  | 17、卫生、社会保障、福利业 |
|  | 18、文化、体育和娱乐业 |
|  | 19、公共管理和社会组织 |
|  | 20、国际组织 |
|  | 21、其他 |

| H14 | 您是医疗保健行业从业人员吗？例如护士、医师、社区卫生工作者或药剂师？**[SR]** |
| --- | --- |
|  | 1、不，我不是医疗保健行业从业人员 |
|  | 2、护士 |
|  | 3、医师 |
|  | 4、社区卫生工作者 |
|  | 5、药剂师 |
|  | 6、其他医疗保健行业从业人员，请说明： |

| H15 | 你的民族是？请选择符合的选项。**[SR]** |
| --- | --- |
|  | 1、汉族 |
|  | 2、回族 |
|  | 3、藏族 |
|  | 4、壮族 |
|  | 5、满族 |
|  | 6、其他，请说明 |

| H16 | 您的家庭总年收入（人民币）是多少？**[SR]** |
| --- | --- |
|  | 1、 |
|  | 2、30000-60000 |
|  | 3、60000-90000 |
|  | 4、90000-120000 |
|  | 5、120000-150000 |
|  | 6、150000-200000 |
|  | 7、200000+ |

| H17 | 请根据您对以下表述的赞同程度进行选择，数字从1到5表示赞同程度递增**[MA]** | | | | | |
| --- | --- | --- | --- | --- | --- | --- |
|  |  | 非常不同意 | 不同意 | 中立 | 同意 | 非常同意 |
|  | 1、如果我感染了新型冠状病毒，我的健康将受到严重损害 |  |  |  |  |  |
|  | 2、我认为新型冠状病毒比流感更严重 |  |  |  |  |  |
|  | 3、即使我患上另一种疾病，我也不会去医院，因为我有在医院感染新型冠状病毒的风险 |  |  |  |  |  |
|  | 4、新型冠状病毒将对我的社区造成严重损害 |  |  |  |  |  |
|  | 5、我比其他人更容易感染新型冠状病毒 |  |  |  |  |  |
|  | 6、我相信我能抵御新型冠状病毒 |  |  |  |  |  |
|  | 7、我相信我能比其他人更好地保护自己免受新型冠状病毒的侵害 |  |  |  |  |  |
|  | 8、我害怕新型冠状病毒 |  |  |  |  |  |
|  | 9、新型冠状病毒严重损害了经济 |  |  |  |  |  |
