# Supplementary material for: Attitudes Toward the Global Allocation of Chinese COVID-19 Vaccines: Cross-sectional Online Survey of Adults Living in China
Source: JMIR Public Health Surveill. 2022 Jun 7;8(6):e33484. doi: 10.2196/33484 (PMC9177168; doi:10.2196/33484)
Supplement: Multimedia Appendix 2 [file publichealth_v8i6e33484_app2.docx]

**Multimedia Appendix 2. Supplementary material.**

| **Table A1. Sociodemographic characteristics of the survey participants** | | | |
| --- | --- | --- | --- |
|  | **Survey participants** | | **Population of China^b^** |
| **Characteristic** | ***Proportion*** | ***N (%)*** | ***Proportion*** |
|  | *(weighted)^a^* | *(not weighted)* |  |
| ***Sex*** |  |  |  |
| *Female* | 48.9% | 4921 (49.2) | 48.9% |
| ***Age group*** |  |  |  |
| *< 20 Years* | 10.1% | 900 (9.0) | 21.9% |
| *20-29 Years* | 17.2% | 1645 (16.5) | 13.1% |
| *30-39 Years* | 17.4% | 1895 (19.0) | 15.7% |
| *40-49 Years* | 20.3% | 1890 (18.9) | 15.8% |
| *50-59 Years* | 17.7% | 1820 (18.2) | 15.3% |
| *> 60 Years* | 17.3% | 1850 (18.5) | 18.1% |
| ***Education*** |  |  |  |
| *Never Been to School* | 5.0% | 526 (5.3) | 5.1% |
| *Elementary School* | 4.9% | 537 (5.4) | 25.3% |
| *Middle School* | 17.1% | 1753 (17.5) | 37.3% |
| *High School / Technical Secondary School* | 36.2% | 3512 (35.1) | 17.7% |
| *College / Undergraduate* | 34.2% | 3371 (33.7) | 13.9% |
| *Graduate and Above* | 2.6% | 301 (3.0) | 0.6% |
| ***Ethnicity*** |  |  |  |
| *Han* | 95.3% | 9444 (94.4) | 95.0% |
| *Man* | 0.7% | 125 (1.3) | 0.7% |
| *Hui* | 0.1% | 52 (0.5) | 0.8% |
| *Zang* | 1.5% | 130 (1.3) | 0.5% |
| *Zhuang* | 1.2% | 128 (1.3) | 1.2% |
| *Other* | 1.3% | 121 (1.2) | 1.8% |
| ***Province of current residence*** |  |  |  |
| *Anhui* | 4.5% | 360 (3.6) | 4.5% |
| *Beijing* | 1.5% | 360 (3.6) | 1.5% |
| *Chongqing* | 2.2% | 300 (3.0) | 2.2% |
| *Fujian* | 2.8% | 300 (3.0) | 2.8% |
| *Gansu* | 1.9% | 300 (3.0) | 1.9% |
| *Guangdong* | 8.2% | 360 (3.6) | 8.2% |
| *Guangxi* | 3.5% | 300 (3.0) | 3.5% |
| *Guizhou* | 2.6% | 300 (3.0) | 2.6% |
| *Hainan* | 0.7% | 300 (3.0) | 0.7% |
| *Hebei* | 5.4% | 360 (3.6) | 5.4% |
| *Heilongjiang* | 2.7% | 300 (3.0) | 2.7% |
| *Henan* | 6.9% | 360 (3.6) | 6.9% |
| *Hubei* | 4.2% | 300 (3.0) | 4.2% |
| *Hunan* | 4.9% | 300 (3.0) | 4.9% |
| *Jiangsu* | 5.8% | 360 (3.6) | 5.8% |
| *Jiangxi* | 3.3% | 300 (3.0) | 3.3% |
| *Jilin* | 1.9% | 300 (3.0) | 1.9% |
| *Liaoning* | 3.1% | 340 (3.4) | 3.1% |
| *Neimengol* | 1.8% | 300 (3.0) | 1.8% |
| *Ningxia* | 0.5% | 300 (3.0) | 0.5% |
| *Qinghai* | 0.4% | 300 (3.0) | 0.4% |
| *Shaanxi* | 2.8% | 300 (3.0) | 2.8% |
| *Shandong* | 7.2% | 360 (3.6) | 7.2% |
| *Shanghai* | 1.7% | 360 (3.6) | 1.7% |
| *Shanxi* | 2.7% | 300 (3.0) | 2.7% |
| *Sichuan* | 6.0% | 360 (3.6) | 6.0% |
| *Tianjin* | 1.1% | 360 (3.6) | 1.1% |
| *Tibet* | 0.3% | 300 (3.0) | 0.3% |
| *Xinjiang* | 1.8% | 300 (3.0) | 1.8% |
| *Yunnan* | 3.5% | 300 (3.0) | 3.5% |
| *Zhejiang* | 4.2% | 360 (3.6) | 4.2% |
| ***Rural-urban residency*** |  |  |  |
| *Urban* | 61.1% | 5935 (59.4) | 60.6% |
| ***Works as a Health Care Provide****r* |  |  |  |
| *No* | 98.3% | 9829 (98.3) | 99.1% |
| *Nurse* | 0.4% | 35 (0.4) | 0.3% |
| *Physician* | 0.5% | 46 (0.5) | 0.3% |
| *Community Health Worker* | 0.4% | 51 (0.5) | <0.1% |
| *Pharmacist* | 0.1% | 13 (0.1) | <0.1% |
| *Other Healthcare Provider* | 0.3% | 26 (0.3) | 0.2% |
| ***Annual Household Income (RMB****)* |  |  |  |
| *< 30,000* | 6.1% | 572 (5.72) | - |
| *30,000 - 59,999* | 13.4% | 1307 (13.07) | - |
| *60,000 - 89,999* | 20.5% | 1929 (19.29) | - |
| *90,000 - 119,999* | 18.0% | 1726 (17.26) | - |
| *120,000 - 149,999* | 16.7% | 1726 (17.26) | - |
| *150,000 - 199,999* | 16.8% | 1882 (18.82) | - |
| *≥ 200,000* | 8.6% | 858 (8.6) | - |

^a^Weighted using survey sampling weights.

^b^As per the 2020 China Statistical Yearbook

**Table A2. Multivariale regression** **results of attitude variation by sociodemographic characteristics**^a^

| **Characteristics** | **Supporting COVID-19 vaccines provision to foreign countries before fulfilling all domestic needs**^b^ | ***P* value** | **Supporting COVID-19 vaccines as low-priced or free global public goods^c^** | ***P* value** |
| --- | --- | --- | --- | --- |
| ***Sex*** | | | | |
| Male | 1 (ref.) |  | 1 (ref.) |  |
| Female | 1.17 (1.05 - 1.30) | .005 | 1.08 (0.97 - 1.20) | .17 |
| ***Age group*** | | | | |
| 18-19 years | 1 (ref.) |  | 1 (ref.) |  |
| 20-29 years | 0.78 (0.61 - 1.01) | .06 | 1.05 (0.83 - 1.32) | .68 |
| 30-39 years | 0.62 (0.48 - 0.80) | <.001 | 1.15 (0.91 - 1.46) | .25 |
| 40-49 years | 0.65 (0.52 - 0.82) | <.001 | 1.04 (0.83 - 1.29) | .75 |
| 50-59 years | 0.69 (0.54 - 0.88) | .003 | 0.86 (0.68 - 1.08) | .20 |
| >60 years | 0.60 (0.45 - 0.80) | .001 | 0.86 (0.64 - 1.15) | .32 |
| ***Annual household income (RMB)*** | | | | |
| < 30,000 | 1 (ref.) |  | 1 (ref.) |  |
| 30,000 - 59,999 | 1.33 (1.03 - 1.72) | .03 | 0.75 (0.58 - 0.96) | .02 |
| 60,000 - 89,999 | 1.30 (1.02 - 1.67) | .04 | 0.76 (0.60 - 0.97) | .03 |
| 90,000 - 119,999 | 1.45 (1.12 - 1.87) | .004 | 0.67 (0.52 - 0.86) | .002 |
| 120,000 - 149,999 | 1.46 (1.13 - 1.90) | .004 | 0.69 (0.54 - 0.89) | .004 |
| 150,000 - 199,999 | 1.39 (1.07 - 1.80) | .01 | 0.60 (0.46 - 0.78) | <.001 |
| ≥ 200,000 | 1.29 (0.96 - 1.73) | .09 | 0.62 (0.46 - 0.83) | .001 |
| ***Education*** | | | | |
| Never been to school | 1 (ref.) |  | 1 (ref.) |  |
| Elementary school | 0.79 (0.57 - 1.10) | .16 | 1.10 (0.78 - 1.54) | .60 |
| Middle school | 0.89 (0.66 - 1.21) | .46 | 0.94 (0.69 - 1.29) | .70 |
| High school / Technical secondary school | 0.85 (0.61 - 1.17) | .31 | 0.92 (0.66 - 1.28) | .61 |
| College / Undergraduate | 0.88 (0.63 - 1.22) | .44 | 0.76 (0.53 - 1.07) | .12 |
| Graduate and Above | 1.08 (0.68 - 1.73) | .73 | 0.83 (0.52 - 1.33) | .44 |
| ***Rural-urban residency*** | | | | |
| Rural | 1 (ref.) |  | 1 (ref.) |  |
| Urban | 0.88 (0.79 - 0.99) | .03 | 0.93 (0.83 - 1.04) | .21 |
| ***Work as a healthcare provider*** | | | | |
| No | 1 (ref.) |  | 1 (ref.) |  |
| Nurse | 2.27 (0.82 - 6.28) | .11 | 1.77 (0.81 - 3.87) | .15 |
| Physician | 0.96 (0.41 - 2.27) | .93 | 1.23 (0.55 - 2.74) | .61 |
| Community health worker | 1.01 (0.47 - 2.19) | .98 | 1.34 (0.65 - 2.75) | .43 |
| Pharmacist | 1.12 (0.03 - 4.23) | .87 | 1.35 (0.38 - 4.84) | .65 |
| Other healthcare provider | 1.39 (0.48 - 4.03) | .54 | 0.84 (0.32 - 2.20) | .72 |
| ***Knows someone with a confirmed SARS-CoV-2 infection*** | | | | |
| No | 1 (ref.) |  | 1 (ref.) |  |
| Self | 1.40 (0.11 - 18.51) | .80 | 0.00 (0.00 - 0.00) | <.001 |
| Family member | 0.53 (0.05 - 5.48) | .59 | 0.00 (0.00 - 0.00) | <.001 |
| Friend | 0.99 (0.20 - 4.99) | .99 | 1.52 (0.30 - 7.62) | .61 |
| Neighbor | 0.89 (0.07 - 11.20) | .93 | 0.00 (0.00 - 0.00) | <.001 |
| Coworker | 0.06 (0.01 - 0.66) | .02 | 2.86 (0.32 - 25.71) | .35 |
| Others | 6.79 (0.84 - 55.22) | .07 | 0.00 (0.00 - 0.00) | <.001 |

^a^ Regression included all of the variables (sex, age group, income, education, rural-urban residency, vocation, whether or not a participant has a family member, friend, or acquaintance who they know to have been infected with SARS-CoV-2) shown in the table and a binary indicator for each province (province-level fixed effects).

^b^ “Satisfy the vaccination needs of all Chinese people before providing to others” is the reference response.

^c^ “At market price or a small profit” is the reference response.

**Table A3. Ordered logistic regression of attitude variation by sociodemographic characteristics**^a^

| **Characteristics** | **Beta of attitudes towards COVID-19 vaccine distribution priorities^b,c^** | ***P* value** | **Beta of attitude towards price of the COVID-19 vaccines provided to foreign countries^b,d^** | ***P* value** |
| --- | --- | --- | --- | --- |
| ***Sex*** | | | | |
| Male | 0 (ref.) |  | 0 (ref.) |  |
| Female | -0.13 | <.001 | -0.01 | .83 |
| ***Age group*** | | | | |
| 18-19 years | 0 (ref.) |  | 0 (ref.) |  |
| 20-29 years | 0.07 | .24 | -0.09 | .12 |
| 30-39 years | 0.19 | .001 | 0.00 | .98 |
| 40-49 years | 0.19 | .001 | -0.06 | .31 |
| 50-59 years | 0.16 | .005 | -0.15 | .008 |
| >60 years | 0.19 | .005 | -0.13 | .05 |
| ***Annual household income (RMB)*** | | | | |
| < 30,000 | 0 (ref.) |  | 0 (ref.) |  |
| 30,000 - 59,999 | -0.11 | .12 | -0.07 | .33 |
| 60,000 - 89,999 | -0.06 | .35 | -0.06 | .38 |
| 90,000 - 119,999 | -0.09 | .18 | -0.06 | .33 |
| 120,000 - 149,999 | -0.07 | .30 | -0.09 | .17 |
| 150,000 - 199,999 | -0.10 | .13 | -0.10 | .15 |
| ≥ 200,000 | -0.06 | .44 | -0.10 | .19 |
| ***Education*** | | | | |
| Never been to school | 0 (ref.) |  | 0 (ref.) |  |
| Elementary school | 0.08 | .35 | 0.06 | .46 |
| Middle school | 0.05 | .54 | -0.06 | .41 |
| High school / Technical secondary school | 0.06 | .43 | -0.10 | .20 |
| College / Undergraduate | 0.09 | .30 | -0.14 | .081 |
| Graduate and Above | -0.07 | .52 | -0.13 | .23 |
| ***Rural-urban residency*** | | | | |
| Rural | 0 (ref.) |  | 0 (ref.) |  |
| Urban | 0.11 | <.001 | -0.03 | .29 |
| ***Work as a healthcare provider*** | | | | |
| No | 0 (ref.) |  | 0 (ref.) |  |
| Nurse | -0.20 | .42 | 0.27 | .19 |
| Physician | -0.23 | .30 | 0.03 | .90 |
| Community health worker | -0.11 | .59 | 0.13 | .43 |
| Pharmacist | -0.02 | .94 | 0.23 | .49 |
| Other healthcare provider | 0.00 | .99 | -0.01 | .97 |
| ***Knows someone with a confirmed SARS-CoV-2 infection*** | | | | |
| No | 0 (ref.) |  | 0 (ref.) |  |
| Self | -0.01 | .97 | -4.06 | <.001 |
| Family member | 0.26 | .56 | -2.10 | .26 |
| Friend | 0.37 | .12 | 0.04 | .93 |
| Neighbor | 0.18 | .66 | -4.05 | <.001 |
| Coworker | 1.84 | .01 | 0.94 | .04 |
| Others | -0.57 | .12 | -0.20 | .55 |

^a^ Regression included all of the variables (sex, age group, income, education, rural-urban residency, vocation, whether or not a participant has a family member, friend, or acquaintance who they know to have been infected with SARS-CoV-2) shown in the table and a binary indicator for each province (province-level fixed effects).

^b^ Dependent variables were included in the model as original responses without recoding. The order of dependent variables is consistent with the order of options in the original questionnaire.

^c^ Q4 with a rank of 1 have the highest willingness to provide COVID-19 vaccines to foreign countries, a rank of 4 have the lowest.

^d^ Q9 with a rank of 1 have the highest price, a rank of 5 have the lowest.


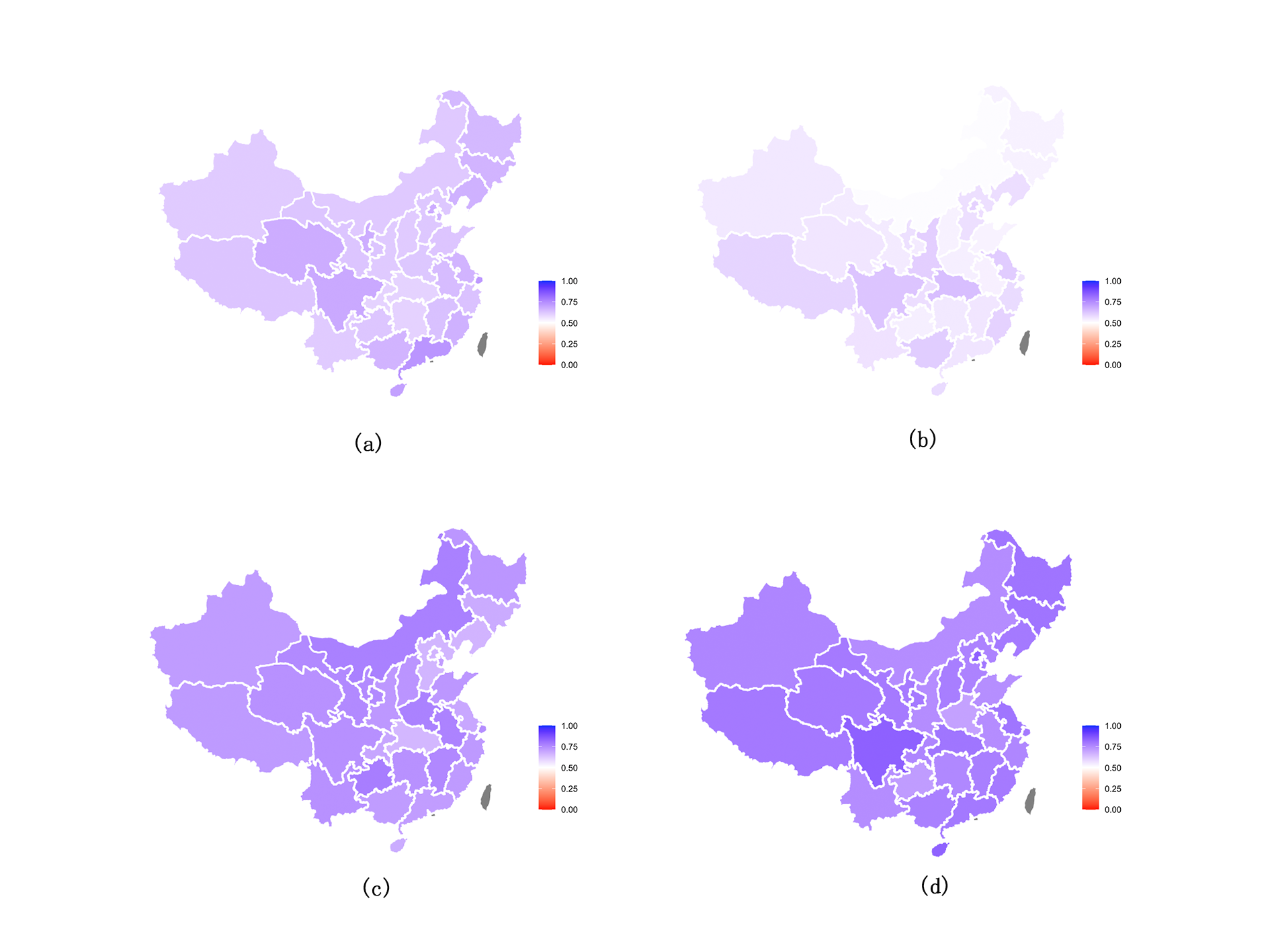


**Figure A1. The proportion of the population by province* (a) supporting that providing COVID-19 vaccines developed by Chinese scientific research institutions and enterprises is the preferable ways to provide assistance to foreign countries; (b) friendly countries that have diplomatic relations with China should they be first provided to, if COVID-19 vaccines developed by Chinese scientific research institutions and enterprises are provided to foreign countries; (c) To provide financial support via authoritative international organizations or specialized organizations is the most preferable if China provides financial supports to foreign countries; and (d) To provide finished vaccine products directly to foreign countries, or to transfer vaccine technology to relevant countries to allow local production, is the most preferable if China provides COVID-19 vaccines to foreign countries.**
